# Genome Sequences for a Cluster of Human Isolates of *Listeria monocytogenes* Identified in South Africa in 2015

**DOI:** 10.1128/genomeA.00200-16

**Published:** 2016-04-07

**Authors:** Anthony M. Smith, Preneshni Naicker, Colleen Bamford, Liliwe Shuping, Kerrigan M. McCarthy, Arvinda Sooka, Shannon L. Smouse, Nomsa Tau, Karen H. Keddy

**Affiliations:** a Centre for Enteric Diseases, National Institute for Communicable Diseases, Johannesburg, South Africa; b Faculty of Health Sciences, University of the Witwatersrand, Johannesburg, South Africa; c National Health Laboratory Service (Groote Schuur Hospital), Cape Town, South Africa; d Division of Public Health Surveillance and Response, National Institute for Communicable Diseases, Johannesburg, South Africa

## Abstract

*Listeria monocytogenes* is a Gram-positive bacterium with a ubiquitous presence in the environment. There is growing concern about the increasing prevalence of *L. monocytogenes* associated with food-borne outbreaks. Here we report genome sequences for a cluster of human isolates of *L. monocytogenes* identified in South Africa in 2015.

## GENOME ANNOUNCEMENT

*Listeria monocytogenes* is a Gram-positive bacterium causing the disease listeriosis. The bacterium has a ubiquitous presence in the environment. Although listeriosis is relatively rare, infections can have fatality rates as high as 30% (1). *L. monocytogenes* is an opportunistic pathogen. Acquisition of the pathogen occurs mainly by consumption of contaminated food. Infections with *Listeria* can result in mild febrile gastroenteritis in healthy individuals; however, invasive disease characterized by bacteremia, meningitis, pneumonia, endocarditis, and sepsis can occur in high risk groups (2). The pathogen most commonly affects immunocompromised individuals, pregnant women, neonates, and the elderly. There is growing concern about the increasing prevalence of *L. monocytogenes* associated with food-borne outbreaks (3, 4). Reports, publications and data concerning the prevalence and epidemiology *L. monocytogenes* in South Africa are lacking. To prevent, investigate, and control outbreaks of disease, it is vital to have information about the molecular epidemiology of the disease. In particular, genomic sequence data can be used to investigate the population structure and evolution of pathogens (5). In the present study, we describe genomic sequence data which will greatly contribute to the current limited epidemiological data that exists for *L. monocytogenes* in South Africa.

During September 2015, an increased number of human cases of *L. monocytogenes* were isolated from hospitals in the Western Cape Province of South Africa. Among the cases were 3 isolates of *L. monocytogenes*, all recovered from blood culture specimens. One patient was a pregnant woman (24 years old), while the two other patients were neonates (1 day old). All *L. monocytogenes* isolates were later determined to belong to multilocus sequence typing (MLST) subtype ST6, a subtype commonly associated with unfavorable outcomes in patients (6).

Whole-genome sequencing analysis of the bacterial isolates was completed at the Centre for Enteric Diseases of the National Institute for Communicable Diseases. Genomic DNA was isolated from bacteria using the Qiagen QIAamp DNA minikit (Qiagen, Hilden, Germany). DNA libraries were prepared using a Nextera XT DNA library preparation kit (Illumina, San Diego, CA, USA), followed by a 2 × 300 paired-end sequencing runs with 100× coverage using Illumina MiSeq equipment. Raw data generated on the MiSeq was further analyzed using tools available in the CLC Genomics Workbench Software, version 8.5 (Qiagen). Using the “Trim Sequences Tool,” sequence reads were trimmed to include quality trimming and ambiguity trimming, and length trimming to discard reads below a length of 100 bases. Trimmed reads were assembled using the “*De novo* Assembly Tool”; the assembly algorithm works by using de Bruijn graphs to produce contiguous (contig) sequences (minimum contig length was set at 500 bases). For the assemblies, final contig numbers ranged from 48 to 85, with *N*_50_ contig values ranging from 150,269 to 184,970. Contig measurements estimated genomes sizes of ~3 Mb with G+C nucleotide content of 38%.

### Nucleotide sequence accession numbers.

This whole-genome shotgun project has been deposited at DDBJ/EMBL/GenBank under the accession numbers LROO00000000, LROP00000000, and LROQ00000000. The versions described in this paper are the first versions, LROO01000000, LROP01000000, and LROQ01000000.
